# The Dynamic Regulation of mRNA Translation and Ribosome Biogenesis During Germ Cell Development and Reproductive Aging

**DOI:** 10.3389/fcell.2021.710186

**Published:** 2021-11-03

**Authors:** Marianne Mercer, Seoyeon Jang, Chunyang Ni, Michael Buszczak

**Affiliations:** Department of Molecular Biology, The University of Texas Southwestern Medical Center, Dallas, TX, United States

**Keywords:** mRNA translation, ribosome, germline, aging, development, ribosome biogenesis

## Abstract

The regulation of mRNA translation, both globally and at the level of individual transcripts, plays a central role in the development and function of germ cells across species. Genetic studies using flies, worms, zebrafish and mice have highlighted the importance of specific RNA binding proteins in driving various aspects of germ cell formation and function. Many of these mRNA binding proteins, including Pumilio, Nanos, Vasa and Dazl have been conserved through evolution, specifically mark germ cells, and carry out similar functions across species. These proteins typically influence mRNA translation by binding to specific elements within the 3′ untranslated region (UTR) of target messages. Emerging evidence indicates that the global regulation of mRNA translation also plays an important role in germ cell development. For example, ribosome biogenesis is often regulated in a stage specific manner during gametogenesis. Moreover, oocytes need to produce and store a sufficient number of ribosomes to support the development of the early embryo until the initiation of zygotic transcription. Accumulating evidence indicates that disruption of mRNA translation regulatory mechanisms likely contributes to infertility and reproductive aging in humans. These findings highlight the importance of gaining further insights into the mechanisms that control mRNA translation within germ cells. Future work in this area will likely have important impacts beyond germ cell biology.

## Introduction

Germ cells are essential for the propagation of multicellular species and share many features that have long fascinated biologists. They undergo extensive epigenetic reprogramming back to a state that supports totipotency in the fertilized zygote, they are exceptionally good at repairing DNA damage, they are the only cells in our bodies that undergo meiosis, and they protect against the invasion and proliferation of transposable elements. Germ cells also spend portions of their life in a transcriptionally quiescent state, necessitating the use of translation-based mechanisms to achieve changes in their gene expression programs. Indeed, genetic studies in *Drosophila*, *Caenorhabditis elegans*, zebrafish, and mice have demonstrated that stage-specific regulation of mRNA translation, both at the level of individual transcripts and on a global scale, plays a central role in the formation, differentiation, and function of germ cells across species. This review will be divided into two main sections. The first will focus on how RNA binding proteins influence the translation of key regulators of germ cell formation and female germ cell differentiation. Many of these RNA binding proteins bind to elements within the 3′UTR of target transcripts and directly or indirectly interfere with cap dependent translation initiation. Through the course of this first section, we will also touch upon emerging models of *in trans* regulation between mRNAs and how condensate formation may influence translation of individual transcripts. The second section will be devoted to discussing how ribosome biogenesis, ribosomal protein paralogs, global regulation of translation, and ribosome storage impact germ cell function. The dynamic regulation of ribosome biogenesis and global protein synthesis represents a relatively new and underexplored theme in the context of germ cell development. As such, we will highlight key questions that remain in this area.

## RNA Binding Proteins and the Regulated Translation of mRNAs During Germ Cell Formation

**FIGURE 1:**
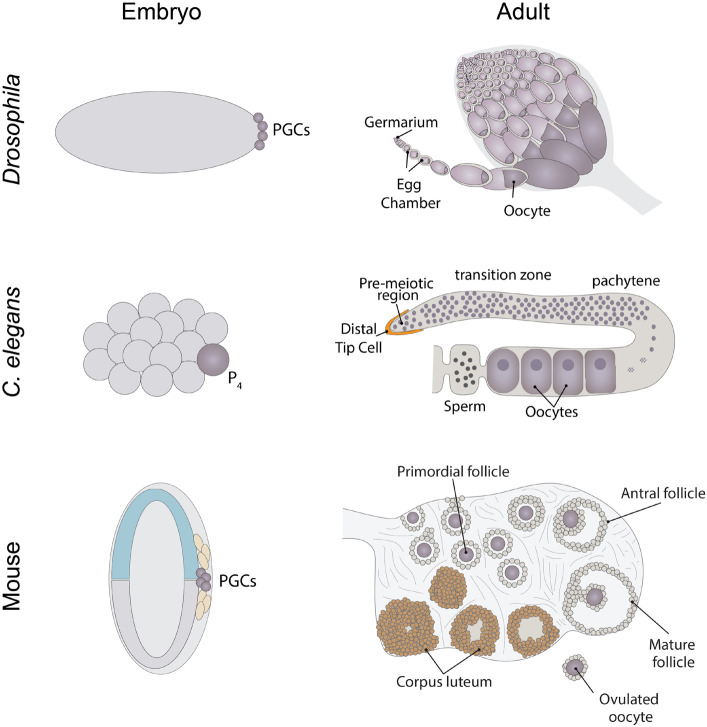
Schematics of where *Drosophila*, *Caenorhabditis elegans*, and mouse PGCs originate from and adult ovary structures.

Genetic studies in a variety of model systems (Figure 1) have led the way in establishing our current understanding of how RNA binding proteins and the regulated translation of individual mRNAs drive various aspects of germ cell development and early embryogenesis. Many of these proteins, including Nanos, Pumilio, Vasa, and Dazl, have long served as useful markers of germ cell identity across different species (Lesch and Page, 2012) (Table 1). These proteins play central roles in establishing germ cell identity, regulating germ cell differentiation, preparing germ cells for entry into meiosis and controlling other aspects of germ cell function. In the interest of space, we will primarily focus on how the regulated translation of individual mRNAs controls the specification and differentiation of *Drosophila* female germ cells. Importantly, many excellent recent reviews describe important findings from other model systems (Voronina et al., 2011; Lai and King, 2013; Susor et al., 2016; Jamieson-Lucy and Mullins, 2019; Huggins and Keiper, 2020). We will touch upon commonalities in the regulation of translation between these different species.

**TABLE 1:** Germ cell enriched genes across species.

	*Caenorhabditis elegans*	*Drosophila*	Zebrafish	Mammals	Function
Vasa	GLH-1, GLH-2, GLH-3, GLH-4	Vasa	DDX4	DDX4	RNA helicase; Polar granule component; Positive regulation of mRNA translation
Nanos	NOS-1, NOS-2	Nanos	NANOS-1 NANOS-2 NANOS-3	NANOS-1 NANOS-2 NANOS-3	Zinc finger RNA binding protein; Negative regulation of mRNA translation
Pumilio	FBF-1, FBF-2, PUF-3, PUF-5, PUF-6, PUF-7, PUF-9, PUF-11	Pumilio	PUM1, PUM2	PUM1, PUM2	RNA binding protein; Negative regulation of mRNA translation; Linked with inhibition of meiosis
Dazl	DAZ-1	Boule	DAZL	DAZ, DAZL, BOULE	RNA binding protein; Negative and positive regulation of mRNA translation; Promotes progression through meiosis

### Germ Plasm Formation in *Drosophila*

**FIGURE 2:**
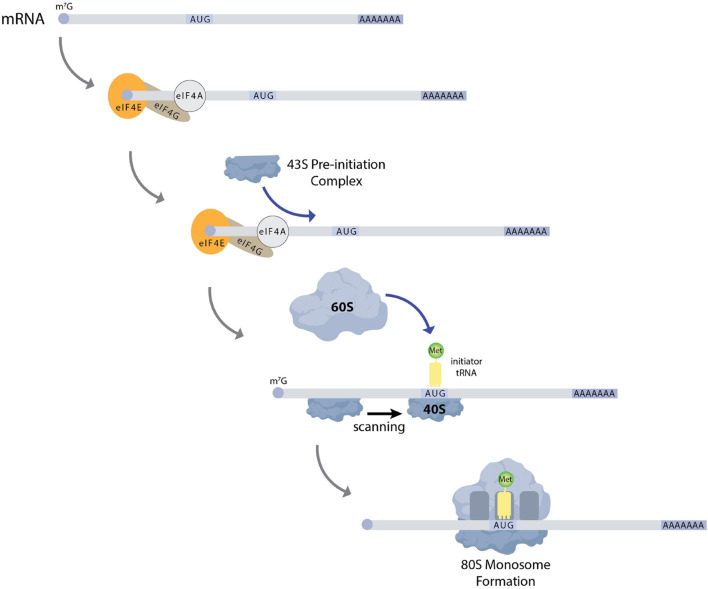
Schematic of general translation initiation. mRNAs generally have a 7-methylguanosine cap and a poly-A tail. The cap serves to recruit the translation initiation factor eIF4E to mRNAs. eIF4E then recruits the scaffold protein eIF4G, which in turn brings the RNA helicase eIF4A to mRNA. eIF4A serves to unwind secondary structure within the 5′UTR of mRNAs, which allows for the recruitment of the 43S pre-initiation complex. This complex then scans the mRNA for a start codon, typically AUG. Next the 60S subunit is recruited to the mRNA to form a 80S monosome. Multiple ribosomes can associate with mRNAs to form polysomes.

Characterization of *Drosophila* germ cell formation has served as a useful platform for understanding how the regulation of mRNA translation initiation (Figure 2) impacts cell fate decisions [reviewed in Lehmann (2016)]. While we have gained substantial knowledge regarding many of the RNA binding proteins involved in controlling germ cell specification and development, several important questions remain: how do different regulatory mechanisms coordinate with one another, how is the translation of specific messages turned on and off in a temporally specific manner, and to what extent does condensate formation and phase transitions allow for the translation of specific messages to be toggled back and forth between an active and repressed state? Reviewing the current knowledge will provide context for discussing these and other open questions in the field.

Early *Drosophila* embryos initially develop as a syncytium in which nuclei undergo mitotic divisions in a common cytoplasm. This cytoplasm is patterned, with RNAs and proteins localized to specific regions within the embryo. Nuclei move to the periphery of the embryo and are eventually surrounded by plasm membrane to form individual cells, through a process known as cellularization. Primordial germ cells form during cellularization in the posterior pole of the embryo through uptake of specialized cytoplasm referred to as germ plasm (Figure 1). Germ plasm formation depends on Oskar protein, which contains two RNA-binding domains: the OSK RNA-binding domain and the OST-HTH/LOTUS domain (Markussen et al., 1995; Rongo et al., 1995; Lehmann, 2016). Oskar mRNA and protein are first localized to the posterior pole of the oocyte during oogenesis. To begin to understand the mechanisms that control *Drosophila* germ plasm formation, we must first consider the process of oogenesis.

**FIGURE 3:**
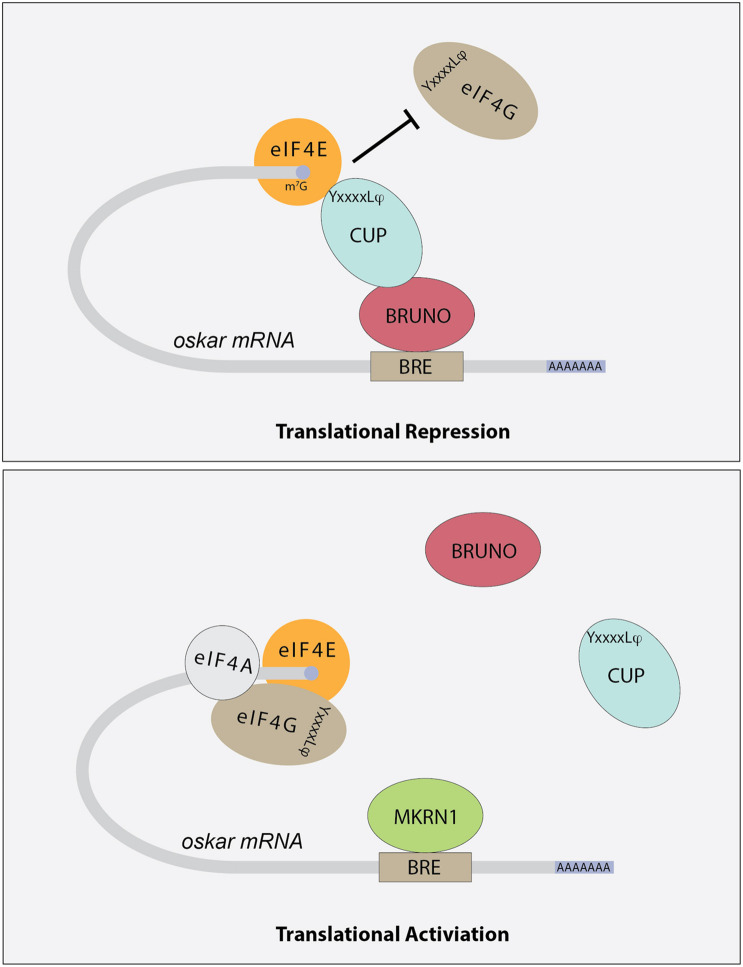
Schematic showing how different factors contribute to the regulation of *oskar* mRNA translation. Bruno binds to Bruno Response Elements (BREs) in the 3′UTR of target mRNAs, including *oskar*. Bruno associates with Cup protein, which in turns binds to eIF4E, preventing the recruitment of eIF4G and effectively blocking translation initiation. The YxxxxLφ (where *x* is any amino acid and φ is a hydrophobic amino acid) motif is indicated. Other mRNA binding proteins, including Mkrn1, compete with Bruno for binding. Once these proteins displace Bruno and Cup, translation initiation can proceed.

*Drosophila* ovaries are composed of tube-like structures called ovarioles, which house sequentially developing egg chambers (Figure 1). Each developing egg chamber contains 16 interconnected germ cells (15 nurse cells and 1 oocyte), surrounded by a layer of somatically derived follicle cells (Spradling, 1993). During most of oogenesis, the oocyte is transcriptionally quiescent. The synthesis of RNAs and many proteins occurs in the nurse cells and these molecules are then actively transported to the oocyte, often in a microtubule dependent manner. The transport of *oskar* mRNA from nurse cells to the oocyte, and its repression during this transport, depends on several proteins including Staufen, components of the Exon Junction Complex and Bruno (Kim-Ha et al., 1991; St Johnston et al., 1991; Hachet and Ephrussi, 2004). Importantly, the translation of *oskar* mRNA is repressed until it localizes to the posterior pole in the oocyte, and then only at a developmentally appropriate time. This repression depends on the mRNA binding protein Bruno. Sequencing the *Drosophila* genome revealed 3 Bruno paralogs: Bruno1, 2, and 3. Bruno1 binds to Bruno Response elements (BREs) within the 3′UTR of *oskar* mRNA through its RNA recognition motifs (Snee et al., 2008). BREs and other regulatory elements cluster within two regions at opposite ends of the *oskar* 3′UTR: the AB region and the C region (Reveal et al., 2010). All three participate in the translational repression of *oskar* mRNA and region C has an additional role in promoting *oskar* translation once the mRNA reaches the posterior pole of the oocyte. Bruno binding to these regions serves to recruit a second protein, Cup (Nakamura et al., 2004) (Figure 3). Cup was originally characterized based on its female sterile phenotype and regulation of nurse cell chromosome morphology (Keyes and Spradling, 1997). Cup resides in the cytoplasm (Keyes and Spradling, 1997), and a collection of studies found that this protein plays an important role in regulating the translation of key transcripts during oogenesis (Verrotti and Wharton, 2000; Wilhelm et al., 2003, 2005; Macdonald, 2004; Nakamura et al., 2004; Nelson et al., 2004; Zappavigna et al., 2004; Piccioni et al., 2005, 2009; Chekulaeva et al., 2006; Clouse et al., 2008; Igreja and Izaurralde, 2011; Wong and Schedl, 2011; Kinkelin et al., 2012; Gotze et al., 2017). Cup contains a YxxxxLφ (where x is any amino acid and φ is a hydrophobic amino acid) motif found in several eIF4E binding proteins, including the translation initiation factor eIF4G (Figure 3). Cup binding to eIF4E associated with the 7-methylguanosine cap blocks eIF4G binding and thus prevents translation initiation (Wilhelm et al., 2003; Nakamura et al., 2004; Nelson et al., 2004; Kinkelin et al., 2012). Packaging of *oskar* mRNA into silencing particles, which prevent mRNAs from engaging with ribosomes may also contribute to the spatial and temporal regulation of *oskar* mRNA translation (Chekulaeva et al., 2006). Traditionally, Bruno and Cup are typically modeled as acting *in cis*. Intriguingly, expression of an *oskar* mRNA engineered to not produce a protein but carrying all the endogenous regulatory elements can rescue the correct regulation of *oskar* mRNAs which have had all their BREs mutated (Reveal et al., 2010). This observation suggests the possibility of additional *in trans* regulation, whereby Bruno binding to one *oskar* transcript influences the regulation of other *oskar* transcripts. In a follow-up study, Macdonald et al. (2016) presented additional transgenic rescue experiments and molecular modeling to provide further support the model of *in trans* regulation of *oskar* mRNA regulation. Importantly, this regulation likely takes place in the context of higher order ribonucleoprotein particles (RNPs). RNPs belong to a group of structures collectively referred to as biomolecular condensates, which represent a local concentration of molecules in membrane-less foci. While liquid-liquid phase separation, defined as process by which liquid phases form to minimize free energy, underlies the formation of many condensates, this is not always the case [reviewed in Lyon et al. (2021)]. How phase separation and condensate formation contributes to the regulation of mRNA translation is an ongoing area of study. An intriguing possibility is that the in *trans* regulation of *oskar* translation reflects the association of co-regulated *oskar* mRNAs with specific condensates. In light of growing evidence that RNAs and RNA binding proteins accumulate in discrete compartments within cells, the further study of *in trans* regulation of mRNA translation and the control of condensate formation will have important implications beyond germ cell specification.

Once *oskar* mRNA localizes to the posterior pole of the oocyte, repression of its translation is relieved. The mechanisms that regulate this switch from a repressed to an active state are coming into focus. For example, over-expression of *oskar* 3′UTR promotes *oskar* translation, suggesting that repressors such as Bruno are expressed at limited levels and may be titrated away from *oskar* mRNA under the right circumstances (Smith et al., 1992; Kanke and Macdonald, 2015). In addition, several factors including the poly-A-binding proteins Orb and PABP are needed for *oskar* translation (Castagnetti and Ephrussi, 2003; Vazquez-Pianzola et al., 2011). More recent results indicate that a member of the Makorin family of proteins, which contain C3H-type zinc fingers and a RING E3 ubiquitin ligase domain, helps to regulate *oskar* translational activation (Dold et al., 2020). *Drosophila* Makorin 1(Mkrn1) has previously been linked with embryonic patterning (Liu and Lasko, 2015), and new results from the Lasko lab provide evidence that this protein promotes pole plasm formation in the oocyte (Dold et al., 2020). Mkrn1 mutants exhibit proper localization of *oskar* mRNA to the posterior pole but do not produce Oskar protein. Mkrn1 itself localizes to the pole plasm and associates with BREs within *oskar* mRNA. More Bruno1 appears to associate with *oskar* mRNA in *Mkrn1* mutants and the Oskar protein defect in *Mkrn1* mutants is partially suppressed by Bruno1 mutations. Together these data support a model whereby Mkrn1 and Bruno1 compete for binding to *oskar* mRNA (Figure 3). Whether the E3 ligase activity of Mrkn1 is needed to promote Oskar protein expression remains an open question. In other contexts, Mrkn1 has also been associated with ribosome-associated translation quality control of polyadenylated transcripts (Hildebrandt et al., 2019). Whether this activity impacts *oskar* translation also remains unknown.

The *oskar* gene encodes short and long protein isoforms (Markussen et al., 1995; Rongo et al., 1995), which serve to recruit a number of other RNA binding proteins to the pole plasm including Vasa (Breitwieser et al., 1996; Jeske et al., 2015). *Drosophila vasa* was originally discovered in a maternal effect lethal screen (Lasko and Ashburner, 1988). *vasa* mutants lack primordial germ cells and have disrupted posterior segments, resulting in embryonic lethality. Vasa is expressed in the germ cells of ovaries and testes, but its function is not required in the testes as male *vasa* mutants are fertile. Several excellent reviews describing Vasa function have been written (Yajima and Wessel, 2011; Lasko, 2013; Wessel, 2016; Dehghani and Lasko, 2017). Briefly, Vasa is a DEAD-box RNA helicase that marks germ cells across species. Vasa activates translation of several mRNAs including *nanos* in the pole plasm of the *Drosophila* embryo (Gavis and Lehmann, 1994). This activity appears to depend on its association with the general translation factor eIF5B (Johnstone and Lasko, 2004), which promotes recruitment of the 60S subunit and formation of 80S monosomes. Thus, Vasa is critical for the translational activation of both components of the pole plasm that help to specify PGCs within developing embryos and for proteins needed for the continued development of germ cells. Beyond regulating translation, Vasa also has additional functions including interacting with components of small RNA surveillance pathways (Xiol et al., 2014).

### The Regulation of *Drosophila* Germ Cell Differentiation by mRNA Translation Based Mechanisms

**FIGURE 4:**
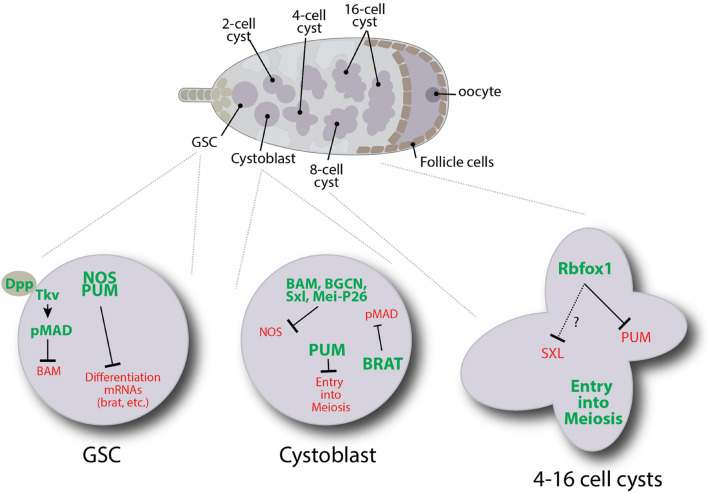
Schematic illustrating some of the translation regulatory pathways that control specific events during the early stages of *Drosophila* female germ cell development.

In addition to germ cell formation, translation regulators are necessary for *Drosophila* germline stem cell (GSC) maintenance and differentiation [reviewed in Slaidina and Lehmann (2014)] (Figure 4). For example, in GSCs, Nanos and Pumilio repress the translation of transcripts that promote differentiation. Nanos belongs to a super family of proteins defined by tandem CCHC zinc fingers (Irish et al., 1989; Wang and Lehmann, 1991; Curtis et al., 1995; Kugler and Lasko, 2009). These zinc fingers interact with RNA. Pumilio is another highly conserved RNA binding protein and is a founding member of the PUF protein family, named for *Drosophila* Pumilio and *C. elegans* fem-3 binding factor (FBF) (Barker et al., 1992; Zamore et al., 1997; Forbes and Lehmann, 1998). Pumilio proteins act to repress mRNA translation and promote the degradation of transcripts. Nanos and Pumilio work together to repress transcripts necessary for differentiation, including *mei-P26* and *brat* (Harris et al., 2011; Joly et al., 2013). It is interesting that while Nanos and Pumilio repress *brat* translation in GSCs, Brat aids Nanos and Pumilio in repressing *hunchback* mRNA in the *Drosophila* embryo (Loedige et al., 2014). These translation repression networks are complex and likely involve many different cofactors. While *mei-P26* and *brat* are known targets, many targets of Nanos and Pumilio have yet to be identified. Target mRNAs of Nanos typically contain Nanos response elements (NREs), which represent the minimal sequence necessary for Nanos mediated repression. Each NRE contains a Pumilio response element (PRE) and is necessary for Nanos and Pumilio to interact (Sonoda and Wharton, 1999). Nanos binds to the three bases upstream of the PRE and acts a clamp, stabilizing the interaction of Pumilio with less favorable PREs (Weidmann et al., 2016). The Nanos N terminus recruits and directly interacts with the CCR4-NOT complex to promote the deadenylation of mRNAs. Target mRNAs are decapped in a deadenylation dependent manner. Nanos can also repress the translation of mRNAs independent of deadenylation, decapping and degradation (Raisch et al., 2016), but the molecular mechanisms that underlie this activity remain uncharacterized.

When GSCs divide, they produce GSCs and cystoblast daughter cells. In GSCs, transcription of the differentiation factor *bam* is repressed by the BMP signaling pathway (Chen and McKearin, 2003a, b; Song et al., 2004; Chen and McKearin, 2005). Any GSC daughter displaced out of the niche no longer receives BMP signals resulting in *bam* expression. Bam is both necessary and sufficient for *Drosophila* germ cell differentiation (McKearin and Spradling, 1990; Ohlstein and McKearin, 1997). Bam works together with Bgcn, Mei-P26 and Sxl to target *nanos* mRNA for repression through a 3′UTR-dependent mechanism (Li et al., 2009). Bam, Bgcn, Mei-P26, and Sxl physically interact, and both Mei-P26 and Sxl interact with *nanos* mRNA (Chau et al., 2012; Li et al., 2012, 2013). Deleting the one Sxl binding site within the 3′UTR of *nanos* prevents its repression (Chau et al., 2012). Yet Sxl alone is not sufficient to repress *nanos* translation as both Sxl and Nanos are expressed in GSCs. In other contexts, Sxl represses translation by interacting with the corepressor Unr, which interacts with PABP to prevent the recruitment of ribosomal preinitiation complexes to 5′UTRs [reviewed in Moschall et al. (2017)]. *nanos* mRNA does not have a clear Unr binding site, suggesting that an alternative mechanism may be at play. Along these lines, Bam appears to recruit the CCR4-NOT complex to promote the decapping and deadenylation of *nanos* and other target mRNAs (Sgromo et al., 2018). Further work will be needed to fully understand the specific roles of Sxl, Bgcn, and Mei-P26 in regulating the decay of target messages.

Unlike Nanos, Pumilio exhibits expression in cystoblasts and two-cell cysts. Pumilio has been shown to form a tertiary complex with Bam and Bgcn through its N terminal domain (Kim et al., 2010). Pumilio also interacts with Brat to repress *mad*, *dMyc*, and components of the BMP signaling pathway (Harris et al., 2011). In addition, Brat associates with Mei-P26 and Ago1 through the NHL domain of Mei-P26 (Neumüller et al., 2008), suggesting these proteins work together to repress factors required for GSC maintenance.

Starting in 4-cell cysts, *pumilio* mRNA translation is repressed by Rbfox1. Loss of Rbfox1 and the continued expression of Pumilio result in developmental arrest (Tastan et al., 2010; Carreira-Rosario et al., 2016). Prolonged expression of Pumilio causes germ cells to dedifferentiate back into a mitotically active state. Rbfox1 belongs to a family of RNA binding proteins that regulate alternative splicing and translation (Conboy, 2017). In *Drosophila* female germ cells, cytoplasmic isoforms of Rbfox1 bind to (U)CGAUG elements in the 3′UTR of *pumilio* transcripts. Interestingly, Rbfox1 does not appear to promote the deadenylation of *pumilio*, and *pumilio* mRNA levels remain unchanged in *Rbfox1* mutants (Carreira-Rosario et al., 2016). The molecular mechanism by which Rbfox1 represses the translation of *pumilio* and its other targets in the germline remains unclear. However, Rbfox1 protein has two intrinsically disordered regions (IDRs) which typically mediate low valency interactions that can promote phase separation (Uversky et al., 2015). Moreover, Rbfox1 colocalizes with several RNP granules suggesting it may promote the sequestration of mRNAs away from translation initiation factors and ribosomes (Kucherenko and Shcherbata, 2018). All together, these various studies indicate that translational regulation represents the major mechanism for regulating gene expression in the *Drosophila* adult female germline. Many RNA binding proteins involved in controlling germ cell differentiation have different binding partners at different stages, adding to the complexity of the regulatory networks. Additional work is needed to understand these networks more fully.

### The Function of *C. elegans* Vasa, Nanos, and Pumilio Homologs

Like *Drosophila*, germ cell formation in *C. elegans* also occurs through a preformation mechanism [reviewed in Wang and Seydoux (2013)]. mRNA-protein complexes called P granules, which are analogous to *Drosophila* germ granules, are distributed throughout the cytoplasm of the one cell embryo (P_0_). The P-granules segregate into cells of the P lineage during the next four divisions. The P_4_ cell then divides to give rise to the germline founder cells called Z2 and Z3, which give rise to the adult germline. P granules continue to be protected from degradation in Z2 and Z3 during subsequent development through to adulthood. Eventually these cells give rise to the adult germline.

*C. elegans* adults can exist as either hermaphrodites or males. The gonad of hermaphrodites has long served as a powerful model for identifying and characterizing factors needed for germline maintenance and function. *C. elegans* adults contain two symmetric U-shaped gonads, which house the germline (Figure 1). Most of the germline exists as a syncytium. Notch signaling from the distal tip cell keeps germline cells in an undifferentiated and proliferative state. As these cells move away from the distal tip, they begin to differentiate and enter meiosis.

Much attention has been given to the characterization of P granules within the *C. elegans* embryonic and adult germline (Wang and Seydoux, 2014). The majority of the protein components of P granules are RNA binding proteins including the Vasa homologs GLH-1, GLH-2, GLH-3, and GLH-4, the P granule assembly factors PGL-1, PGL-2 and PGL-3, and OMA-1 and OMA-2. GLH-1 and GLH-4 function to promote the perinuclear localization of P granules. P granules do not appear to be needed for germ cell specification, but proteins that localize to these structures are needed for fertility. Interestingly, compromising multiple P granule nucleation factors along with GLH-1 and GLH-4 results in the ectopic expression of somatic-specific genes, including factors normally associated with neurons and muscle, within the germline (Updike et al., 2014; Knutson et al., 2017). Recent work has begun to characterize the biophysical properties of P granules (Forman-Kay et al., 2018; Seydoux, 2018; Cable et al., 2019; Lee and Seydoux, 2019; Ouyang et al., 2019; Putnam et al., 2019; Lee et al., 2020; Putnam and Seydoux, 2021). These structures likely represent privileged environments in which resident mRNAs are shielded from engaging with the translation machinery. For example, protein-RNA tethering assays reveal that the translation of reporter mRNAs is repressed upon recruitment to P granules (Aoki et al., 2021). In addition, recent detailed genetic characterization of GLH-1 suggests that Vasa homologs likely serve as “solvents,” which play a variety of important roles within germ cells including promoting the activity of small RNA surveillance pathways and enabling the trafficking of mRNAs out of P granules (Marnik and Updike, 2019; Marnik et al., 2019).

**FIGURE 5:**
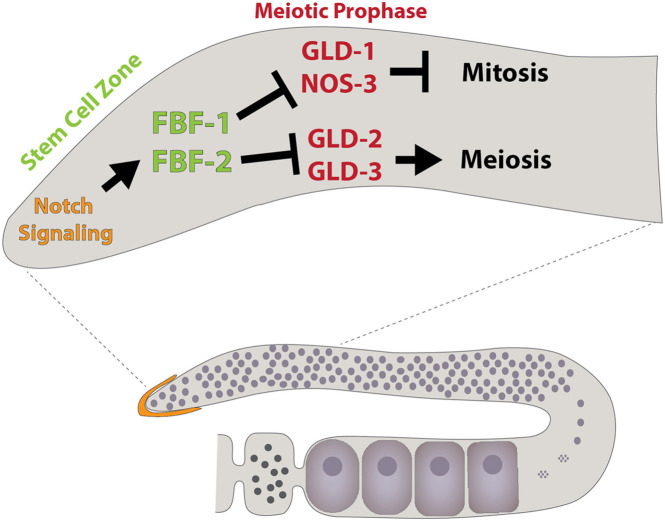
Schematic illustrating some components that regulate *C. elegans* germline development, including the Puf family members FBF-1 and FBF-2. Modeled after Ellenbecker et al. (2019).

The worm genome also encodes 10 Pumilio-like proteins including FBF-1, FBF-2, PUF-8, and PUF-11 [reviewed in Wang and Voronina (2020)]. Half of these genes exhibit enriched expression in germ cells and promote the maintenance of the germline. Initial characterization of FBF-1 and FBF-2 revealed these proteins promote mitotic germline stem cell proliferation (Figure 5). Within this context, both proteins repress the expression of *gld-1*, which drives the commitment to the meiotic cell cycle (Crittenden et al., 2002). Subsequent studies showed the FBF-1 and FBF-2 also repress the expression of multiple components of the synaptonemal complex, the formation of which is a critical step in meiosis and germline differentiation, through a 3′UTR dependent mechanism (Merritt and Seydoux, 2010). The binding of FBF-1 and FBF-2, along with other family members, typically regulate gene expression by deadenylating target mRNAs, resulting in translational repression or RNA decay. In addition, other studies hint at the possibility that worm and mammalian PUF proteins can coordinate with Argonaute miRNA-binding proteins and inhibit translation elongation (Friend et al., 2012).

While earlier work provides evidence that FBF-1 and FBF-2 exhibit functional redundancy (Crittenden et al., 2002; Lamont et al., 2004; Bernstein et al., 2005; Merritt and Seydoux, 2010), significant differences in their mutant phenotypes and subcellular localization have remained poorly understood. Recent papers have begun to resolve this conundrum. FBF-1 appears to restrict the rate at which germline cells enter meiosis, whereas FBF-2 promotes both cell proliferation and entry into meiosis (Wang et al., 2020). Both proteins directly target a common set of mRNAs, including the *Cyclin B* homolog *cyb-2.1*. Mutating FBF binding sites within *cyb-2.1* mRNA and additional loss-of-function experiments provide evidence that the germline coordinates regulation of the cell cycle and meiotic entry through the differential activity of FBF-1 and FBF-2 on specific sets of target genes. Moreover, FBF-1 requires the CCR4-NOT deadenylase complex, while FBF-2 appears to protect messages from deadenylation. These different activities are mediated by regions of the protein outside of the RNA-binding domain.

Additional PUF domain proteins may also contribute to the regulation of the cell cycle and entry into meiosis. Disruption of signaling between the distal tip cell and the germline results in a more severe germline stem cell phenotype than the combined loss of *fbf-1* and *fbf-2* (Austin and Kimble, 1987; Crittenden et al., 2002; Merritt and Seydoux, 2010; Kershner et al., 2014; Shin et al., 2017). Two additional PUF domain proteins, PUF-3 and PUF-11, which play a role in regulating germline maintenance and differentiation have been identified. Genetic analysis reveals that the phenotype of quadruple *fbf-1, fbf-2, puf-3*, and *puf-11* mutants is strikingly similar to glp-1/Notch mutants, revealing new aspects of the complex regulatory PUF protein networks that control germline behavior (Haupt et al., 2020). Further work will be needed to fully delineate how these four *C. elegans* RNA binding proteins coordinate with one another to achieve a balance between germline stem cell divisions and differentiation.

*Caenorhabditis elegans* also express three orthologs of *nanos*, and two of them, *nos-1* and *nos-2*, function in germline development (Subramaniam and Seydoux, 1999). Simultaneous loss of *nos-1* and *nos-2* causes a premature proliferation phenotype in PGCs, resulting in their eventual loss during larval development. Like *Drosophila nanos*, these worm orthologs encode cytoplasmic proteins that target mRNAs for translational silencing or degradation. Nanos-3 physically interacts with the Pumilio homolog FBF-1, and together help to control sperm-oocyte cell fate decisions during development by targeting *fem-3* for post-transcriptional silencing (Kraemer et al., 1999). Subsequent work has focused on defining additional endogenous targets mRNA targets of these three Nanos proteins. The Seydoux lab has shown that loss of *nos-1* and *nos-2* results in both the upregulation of oocyte transcripts and the inappropriate upregulation of other transcripts that are normally kept silent in PGCs (Lee et al., 2017). Interestingly, *nos-1* and *nos-2* appear to repress the expression of LIN-15B, a synMuvB class transcription factor known to antagonize transcriptional silencing. Moreover, disruption of *lin-15b* suppresses both the sterility and the observed changes in the gene expression programs of *nos-1 nos-2* double mutants.

Work in *C. elegans* has also pioneered our understanding of the relationship between translational repression and P granules. Recent work has highlighted the importance of a group of genes called *m*aternal-*e*ffect *g*erm-cell defective (MEG) in controlling P granule formation and activity (Leacock and Reinke, 2008; Kapelle and Reinke, 2011; Smith et al., 2016; Dodson and Kennedy, 2019; Ouyang et al., 2019; Putnam et al., 2019; Lee et al., 2020). For example, proteins that contain intrinsically disordered regions including MEG-2 and MEG-3, promote phase separation (Dodson and Kennedy, 2019; Ouyang et al., 2019; Putnam et al., 2019; Lee et al., 2020). Various mRNAs localize to these condensates, including *nos-2* (Lee et al., 2020). One challenge in the field has been determining whether mRNAs are first recruited to P granules for silencing or whether they accumulate to these granules as a consequence of their repression. Recent experiments examining the regulation of *nos-2* mRNA provide evidence for the latter (Lee et al., 2020). This study found that RNA localization tended to trend with translational status and that accumulation of mRNAs to P granules depended on the activity of translational repressors. Lastly, subjecting worms to heat shock, which disrupts translation initiation, results in a shift of diffusely localized transcripts to P granules (Lee et al., 2020), providing further evidence of links between translational state and localization to P granules.

### Regulation of mRNA Translation by Vasa, Pumilio, and Nanos Homologs in Vertebrate Germ Cells

Multiple studies have identified clear Vasa homologs in zebrafish, *Xenopus*, mice, rats, monkeys and humans (Leroy et al., 1989; Komiya and Tanigawa, 1995; Olsen et al., 1997; Castrillon et al., 2000; Toyooka et al., 2000; Hermann et al., 2007; Mitchell et al., 2008; Encinas et al., 2012; Gassei et al., 2017). These genes have retained their enriched expression in germ cells, and continue to serve as valuable markers of germ cell fate in various contexts [reviewed in Lasko (2013)], including the generation and differentiation of primordial germ cell like cells (Saitou and Miyauchi, 2016; Tang et al., 2016; Kobayashi et al., 2017). The temporal and stage specific expression pattern of Vasa varies from species to species. For example, in mice the expression of DDX4 (the typically used name for Vasa in mammals; also known as Mvh) is first observed in germ cells after they have populated the genital ridge, while in rats DDX4 expression is detectable much earlier in migrating PGCs (Fujiwara et al., 1994; Encinas et al., 2012). In both rodents, DDX4 expression continues in post-meiotic sperm and oocytes. Interestingly, mutations in rodent *DDX4* only appear to disrupt the fertility of males but not females. DDX4 localizes to the chromatoid body, a germ cell specific perinuclear RNA granule, in developing spermatids. Similar to Vasa homologs in *Drosophila* and *C. elegans*, immunoprecipitation experiments show that DDX4 associates with a large number of potential target mRNAs. Some of these mRNAs encode for proteins that play important roles in the translational regulation within the germline, including DDX25 and eIF4B (Tsai-Morris et al., 2004; Nagamori et al., 2011; Yamaguchi et al., 2013).

Homologs of Pumilio and Nanos also contribute to formation, maintenance and development of vertebrate germ cells. Mice and humans have two clear Pumilio homologs, PUM1 and PUM2, and two divergent PUM homologs, PUM3/Puf-A and NOP9 (Goldstrohm et al., 2018). *Pum2* does not appear needed for male or female fertility, although a gene trap within the locus results in morphologically smaller testes (Xu et al., 2007). Deletion of *Pum1* in mice results in reduced male fertility, marked by increased apoptosis in germ cells (Chen et al., 2012). Loss of *Pum1* also leads to subfertility in female mice (Mak et al., 2016), and defects in the maternal phase of embryogenesis (Mak et al., 2018). Subsequent work shows that simultaneous deletion of both *Pum1* and *Pum2* results reduced body size and cell proliferation, partially through mis-regulation of *Cdkn1b* (Lin et al., 2019). Loss of both *Pum1* and *Pum2* also disrupts neurogenesis in mice (Zhang et al., 2017). Like its fly and worm counterparts, PUM1 and PUM2 bind to thousands of transcripts, with significant overlap between the two proteins, in both the testis and nervous system. Similar to other species, these PUM binding sites are enriched for the UGUA(A/C/U)AUA motif. PUM binding to mRNAs typically results in transcript destabilization and/or translational repression. For example, recent work shows that PUM1 forms highly clustered aggregates around *Mad2* and *cyclin B1* RNA granules in mouse oocytes (Takei et al., 2020). This localization correlates with translational repression of these two RNAs. In turn, the breakdown of these PUM1 aggregates correlates with the activation of *Mad2* and *Cyclin B1* translation. Importantly, stabilization of PUM1 aggregates blocks oocyte differentiation, indicating that the dissolution of these aggregates at a particular point in oocyte differentiation is important for their continued maturation.

In related findings, mammalian PUM proteins may also play important roles in the proliferation and differentiation of embryonic stem cells (Uyhazi et al., 2020). Loss of *Pum1* in ESCs results in increased expression of pluripotency genes. By contrast, *Pum2* mutant ESCs display decreased pluripotency and accelerated differentiation. Again, the target mRNAs for both proteins show significant overlap, but within the context of ESCs, PUM1, and PUM2 appear to regulate different subsets of target mRNAs in both a positive and negative manner. In addition, PUM1 and PUM2 regulate the expression of one another, forming regulatory feedback loops.

In addition to co-regulation through 3′UTR-dependent mechanisms, mammals have evolved additional mechanisms for controlling the availability of PUM1 and PUM2. For example, recent work shows that the long non-coding RNA NORAD, which contains a series of PUM binding sites, acts to sequester PUM protein (Lee et al., 2016). Over-expression and increased availability of PUM1 and PUM2 leads to genomic instability in somatic cells, and loss of NORAD in mice results in a striking premature aging phenotype (Kopp et al., 2019). NORAD serves to titrate the amount of available PUM protein to a level that accommodates a cell’s specific needs. New work provides insights into the ability of NORAD to regulate phase transitions through multivalent interactions. Importantly, the formation of NORAD dependent condensates allows for the super-stoichiometric retention of PUM proteins (Elguindy and Mendell, 2021). While *Norad* mutant mice do not appear to have any overt fertility problems, future work will be needed to more thoroughly assay how loss of this and other long non-coding RNAs impact germ cell development and reproductive aging by interacting with translation regulatory machinery. In addition, it will be interesting to test whether germ granules also promote the super-stoichiometric retention of specific mRNAs and their binding proteins.

Mammalian genomes contain three nanos orthologs: *nanos1, nanos2*, and *nanos3* (De Keuckelaere et al., 2018). *nanos1* is expressed in the nervous system and does not appear to function in germ cell development (Haraguchi et al., 2003). By contrast, NANOS2 and NANOS3 play important roles in germ cell maintenance and differentiation (Tsuda et al., 2003; Suzuki et al., 2007). NANOS3 is expressed in primordial germ cells and has served as an important marker in several studies that describe the formation and differentiation of iPS cell and embryonic stem cell derived primordial germ cell-like cells (PGCLCs) (Irie et al., 2015; Irie and Surani, 2017; Chen et al., 2019). *nanos2* is expressed in a male specific manner in spermatogonial stem cells. NANOS2 interacts with Dead end 1 (DND1), another RNA binding protein that promotes the survival of PGCs, and the CCR4-NOT deadenylation complex (Suzuki et al., 2016). Loss of *nanos2* results in reduced expression of DNMT3L, a methyltransferase that functions in establishing male specific DNA methylation patterns. Interestingly, while NANOS2 expression can rescue the germ cell defects caused by disruption of NANOS3, the reverse is not true (Tsuda et al., 2003). A recent study has shed new light on the roles of NANOS2 and NANOS3 in germ cell development (Wright et al., 2021). Double *nanos2* and *nanos3* mutants exhibit a rapid loss of germ cells. NANOS3 serves to prevent apoptosis in germ cells upon loss of *nanos2*. Further analysis shows that while NANOS2 and NANOS3 are structurally similar, the unique amino acid sequence within a zinc finger of NANOS2 is required for its specific interaction with DND1. These biochemical experiments provide a reasonable explanation for why NANOS3 expression cannot rescue *nanos2* mutant phenotypes.

Accumulating evidence shows that orthologs of *vasa, nanos*, and *pumilio* play important roles in the regulation of germ cell formation and function in humans (Jaruzelska et al., 2003; Moore et al., 2003; Lasko, 2013). For example, Pumilio-Nanos complexes function in human germ cells and mutations in *nanos3* have been linked with ovarian insufficiency (Wu et al., 2013; Santos et al., 2014). Interestingly, despite lack of *nanos1* expression in mouse germ cells, mutations in the gene have been linked with human male infertility, marked by oligo-astheno-teratozoospermia or complete germ cell loss (Kusz-Zamelczyk et al., 2013). Human PUM2 protein interacts with two other RNA binding proteins called Deleted in Azoospermia (DAZ) and DAZ-Like (DAZL) (Moore et al., 2003). Encoded by a gene on the Y-chromosome, DAZ has been linked with a number of different defects in human spermatogenesis. The expression of DAZL marks commitment to a germ cell fate and helps to regulate germ cell development and entry into meiosis in mice. A recent paper from the Conti lab provides evidence that DAZL functions to both repress and activate translation of different transcripts within maturing oocytes (Yang et al., 2020). Global analysis shows that ribosome loading onto maternal RNAs is disrupted upon depletion of DAZL. This effect is mediated, in part, through elements found within the 3′UTRs of these transcripts. DAZL directly interacts with these RNAs and phenotypes associated with DAZL loss can be rescued by injection of wildtype DAZL protein. Interestingly, the translation of several transcripts, including *Akap10*, *Cenpe*, *Nsf*, *Ywhaz*, and *Nin*, appears upregulated in the absence of DAZL. Further work shows the directionality of DAZL-dependent regulation depends on other elements found within the 3′UTRs of target mRNAs. This co-regulation was also hinted at in a previous study by the same group (Sousa Martins et al., 2016). The theme of multiple mRNA binding proteins influencing context specific regulation of translation is important across species. Further work will be needed to more fully understand how the presence of multiple factors on individual transcripts is integrated to ensure the proper regulation of translation in space and time. Whether allelic variants of DAZL impact human infertility also requires additional investigation (Rosario et al., 2016).

## Regulation of Translation Machinery During Germ Cell Development

**FIGURE 6:**
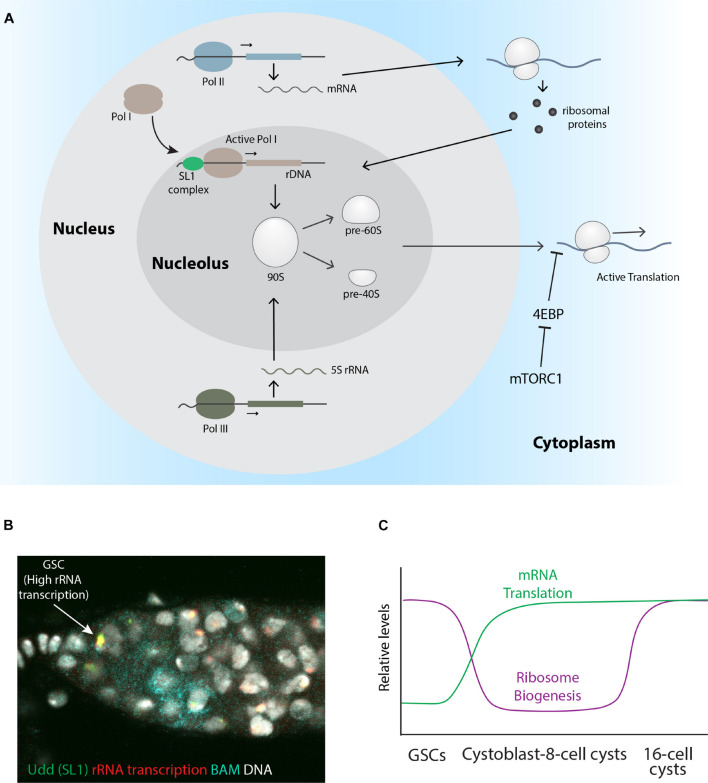
Stage specific regulation of rRNA transcription, ribosome biogenesis, and global protein translation. **(A)** Schematic describing the process of ribosome biogenesis. **(B)** A wildtype *Drosophila* germarium pulse-labeled with Br-UTP to mark nascent rRNA (red) and stained for Udd (green), Bam (cyan), and DNA (gray). The arrow points to a GSC with relatively high rRNA transcription and Udd levels. **(C)** A graph showing the relative levels of ribosome biogenesis and mRNA translation based on published work.

Although previously considered as a house-keeping function, emerging evidence is now showing that protein synthesis can be heterogeneous across different cell-types and developmental stages [reviewed in Buszczak et al. (2014)]. mRNA translation depends on ribosomes, which are composed of about 80 different ribosomal proteins (RPs) and 4 rRNAs. Ribosomes are initially assembled as two distinct subunits, a small 40S subunit and a large 60S subunit within a subdomain of the nucleus called the nucleolus (Klinge and Woolford, 2019) (Figure 6A). Each subunit is independently exported out of the nucleus and into the cytoplasm. Emerging evidence indicates that female germ cells may dynamically regulate ribosome biogenesis during their differentiation as they develop into fertilizable oocytes. Not only is ribosome biogenesis regulated, but specific translation initiation and elongation factors are also enriched and regulated in the germline. Much work still needs to be done to understand how ribosomes and the translation machinery work in concert during the development of the oocyte. Furthermore, oocytes store large numbers of ribosomes for use upon fertilization. Understanding this storage process and how it may deteriorate over time can give us insights into reproductive aging.

### rDNA Repeat Amplification in Germ Cells

rDNA has a direct influence on the total number of ribosomes that can be generated at any point in time. rDNA is commonly organized as tandem repeats, the number of which can vary across species. For example, *Saccharomyces cerevisiae* has ∼150 copies of rDNA on chromosome XII, *Drosophila* has 200–250 repeats on the X and Y chromosomes, and humans have hundreds of rDNA copies in clusters located on multiple chromosomes. rDNA copy number can vary across different mouse strains. In addition, not all repeats are transcribed at any point in time, and we are just beginning to understand the regulatory factors that control the activity of any particular rDNA gene.

Recombination rates within rDNA loci can be relatively high compared to other regions of the genome due to their repetitive nature, leading to increases and decreases in rDNA repeat number (Nelson et al., 2019; Warmerdam and Wolthuis, 2019). rDNA instability increases with age in a range of organisms ranging from yeast to *Drosophila* and rDNA copy number can vary in cancer cells (Wang and Lemos, 2017). Any changes in rDNA repeat number within germ cells will be passed to the next generation and could have a significant impact on the viability and reproductive success of progeny. A recent study adds to the evidence that organisms have evolved mechanisms for maintaining rDNA copy number over multiple generations. Lu et al. (2018) found that aging *Drosophila* males experience a decline in rDNA copy number, which is subsequentially inherited by their offspring. Strikingly, however, germline stem cells from young flies, which initially received a reduced number of rDNA repeats, are able to recover rDNA copy number back to a level more in line with the rest of the population. Thus, it appears that germ cells can “sense” and adjust rDNA copy number, so it is maintained within a species-specific range. What sets this range across species and the mechanisms that underlie this germ cell phenomenon remain unknown, but a recent study using yeast may provide some potential hints (Iida and Kobayashi, 2019). Upstream Activating Factor (UAF) serves to drive Pol I transcription of rRNA. Reduction of rDNA repeats decreases the number of UAF binding sites, in turn leading to increased levels of “free” UAF. UAF, unbound to rDNA, moves to directly repress the histone deacetylase SIR2. SIR2 negatively regulates a number of genes which control recombination rates within the rDNA locus. Reduced expression of these factors results in rDNA amplification. Thus, the movement of UAF from rDNA to the SIR2 gene upon reduction in rDNA copy number, and back to rDNA once copy number has been restored to a level that matches the availability of UAF, represents a simple but elegant feedback loop through which cells can control repeat numbers within this locus. This model predicts that over-expression of UAF may limit viability through multiple cell divisions, as rDNA copy number decreases. Indeed, expressing UAF in yeast strains that already have reduced rDNA copy number results in cell growth defects. Further experiments will be needed to test whether similar mechanisms act within germ cells of multiple cellular organisms to set rDNA copy number within a species-specific range.

In addition to regulating chromosomal rDNA copy number, the oocytes of certain amphibians and fish exhibit the remarkable ability to amplify rDNA by producing extra-chromosomal copies of these repeats. Cytological studies of amphibian oocytes provided the first hints that oocytes may have extra copies of rDNA. Brown and Dawid (1968) extended these earlier studies and found that *Xenopus* germinal vesicles contained hundreds of cresyl violet labeled nucleoli (Brown and Dawid, 1968). Subsequent experiments using equilibrium density gradient centrifugation in CsCl and comparative hybridization between germinal vesicle and somatic cell DNA definitively showed that the oocytes of *Sedum mexicanum* and *Necturus maculosus* contained extra-chromosomal copies of rDNA. Others went on to show that extra-chromosomal rDNA can be found in the oocytes produced by a variety of organisms (Gall, 1968, 1969; Gall et al., 1969; Macgregor, 1972; Gall and Rochaix, 1974; Davidian et al., 2021). Despite their occurrence across several vertebrate species, prevailing evidence indicates that placental mammals do not amplify rDNA within their oocytes using this mechanism (Bachvarova, 1985; Tian et al., 2001).

What is the functional significance of this amplification? Oocytes are often large cells and need a high level of ribosomes to support ongoing mRNA translation over variable periods of storage in the absence of transcription (and hence in the absence of ribosome biogenesis). Many species store massive numbers of ribosomes. Cellular components stored within the oocyte need to accumulate at a sufficient level to support early embryogenesis until the onset of zygotic transcription and the restarting of the ribosome assembly process. In many species, zygotic transcription does not start until after many cell divisions. Thus, changes in gene expression during early differentiation typically occur at the level of mRNA translation. Increased rDNA levels may simply be needed to support the enhanced levels of ribosome biogenesis that occurs in the oocytes of many species. Of note, zygotic transcription in mice and human starts within 1–2 cell divisions after fertilization, perhaps obviating the need for large-scale rDNA amplification.

Knowledge regarding the formation of extra-chromosomal nucleoli comes mostly from the study of amphibian oocytes. A recent paper by Davidian et al. (2021) provides a thoughtful description of the current state of the field. Briefly, the extra synthesis of rDNA begins at the pachytene stage of meiotic prophase, through a gene amplification process. Later, as these oocytes enter the diplotene stage, the rDNA dissociates to form a large number of extra-chromosomal nucleoli. More recent work has characterized the liquid-like properties of these extra-chromosomal nucleoli (Brangwynne et al., 2011; Feric et al., 2016). These findings have paved the way for the further characterization of the biophysical properties of nucleoli and other nuclear bodies from different cell types. Previous studies using electron microscopy suggested that extrachromosomal rDNA may form from rolling circle intermediates (Hourcade et al., 1973, 1974). But what triggers this oocyte-specific gene amplification process during meiosis and how the overall copy number is regulated remains largely unknown. In the future, genetic and biochemical approaches may begin to reveal new insights into this interesting phenomenon.

Other organisms have evolved oocyte specific rRNA genes. For example, *Xenopus* and zebrafish both have oocyte specific 5S rRNAs, the sequence of which differs from their somatic cell counterparts (Wegnez and Monier, 1972; Ford and Southern, 1973; Wakefield and Gurdon, 1983; Locati et al., 2017a). These maternal rRNAs are entirely replaced by a somatic 5S during embryonic development (Wormington and Brown, 1983). This specificity in germline and somatic rRNAs appears to extend to 45S rRNA, the pre-cursor to 5.8S, 18S and 28S rRNAs (Locati et al., 2017b). *In silico* analysis suggests the expansion segments in 18S rRNA may preferentially drive the translation of specific mRNAs in the germline and the soma. More recent experiments, focused on characterizing DNA methylation within the zebrafish germline, uncovered oocyte specific amplification of a 11.5 kb region within the genome that contains 45S rRNA (Ortega-Recalde et al., 2019). Interestingly, the demethylation and amplification of this locus correlates with the expansion of “1B” oocytes. These 1B oocytes contain multiple nucleoli and provide signals that drive the feminization of the gonad. Thus, these results suggest modification of rDNA is linked with sex determination in this species.

### Stage-Specific Regulation of Ribosome Biogenesis During Germ Cell Development

In *Drosophila*, well-conserved growth regulators, such as Myc, modulate female germline growth potential (Maines et al., 2004; Neumüller et al., 2008; Rhiner et al., 2009; Harris et al., 2011). Some studies further suggest that the rate of ribosome production may be different between GSCs and cells within differentiating cysts (Neumüller et al., 2008). For example, during the mitotic divisions of GSCs, Wicked, the *Drosophila* homolog of the U3 snoRNP protein UTP18, becomes enriched in cytoplasmic particles, which asymmetrically segregate to GSCs (Fichelson et al., 2009). snoRNPs contain snoRNAs which serve to guide modification enzymes to specific sites on rRNA, as ribosomes are being assembled in the nucleolus. The asymmetric localization of Wicked suggests that ribosome assembly factors become enriched in GSCs, which in turn support higher levels of ribosome biogenesis in stem cells relative to their differentiating daughters.

This model is also supported by observations that Pol I activity differs between *Drosophila* GSCs and their differentiating progeny (Figure 6B). Across eukaryotes, two functionally distinct Pol I complexes exist: Pol I α and Pol I β. Only Pol I β, which associates with TIF-IA and represents a relatively small fraction of the total soluble Pol I pool, is initiation-competent and capable of productive assembly at the rRNA gene promoter (Miller et al., 2001). In mammalian cells, the selectivity factor 1 (SL1) complex, which consists of TATA-box-binding protein (TBP) and several TBP-associated factors (TAFs), including TAF1B and TAF1C, binds to the core promoter of rDNA genes (Beckmann et al., 1995; Russell and Zomerdijk, 2005; Knutson and Hahn, 2011; Naidu et al., 2011). Once bound, the SL1 complex recruits the TIF-IA-containing Pol I complex to the rDNA promoter (Russell and Zomerdijk, 2005). Components of the *Drosophila* SL1 complex were identified based on the study of a female sterile mutation in a gene called *under-developed* (*udd*). Udd localizes to the nucleolus and is broadly expressed in both germline and somatic cells. Co-staining with various markers revealed that Udd always tightly localizes to a central region within nucleoli of non-dividing cells. Mass spectrometry and co-immunoprecipitation showed that Udd associates with *Drosophila* homologs of the TAF1B and TAF1C Pol I transcription factors. Further genetic experiments show that all three proteins are involved with promoting Pol I transcriptional activity. Pulse-labeling nascent rRNA reveals GSCs exhibit higher levels of rRNA transcription relative to their immediate progeny (Zhang et al., 2014) (Figure 6B). Like Wicked, Udd protein becomes enriched in GSCs immediately after the completion of GSC mitosis, again suggesting that GSCs employ mechanisms to ensure high levels of ribosome production. However, enhanced levels of ribosome biogenesis do not necessarily correlate with high levels of mRNA translation within germ cells. OP-Puro pulse labeling and RNAi knockdown of ribosomal proteins showed that rRNA transcription and protein synthesis are uncoupled during early germ cell differentiation (Sanchez et al., 2016) (Figure 6C). Moreover, ribosome assembly appears to regulate the final steps of mitosis in GSCs. A RNAi knockdown screen in the male germline also revealed a requirement for ribosomal proteins in driving the expression of mitotic factors in GSCs (Liu et al., 2016). These combined results indicate the regulation of ribosome biogenesis and global translation likely influence germ cell development in both males and females.

The developmental potential of vertebrate oocytes may also be closely linked with ribosome biogenesis. Transcriptome analysis of rainbow trout embryo viability indicates that the dynamic regulation of ribosome assembly factors plays a critical role in ensuring egg quality in this species (Ma et al., 2019). In mammals, the nucleolus and the dynamic regulation of rDNA activity plays an essential role in producing fertilizable oocytes [reviewed in Kresoja-Rakic and Santoro (2019)]. During their growth phase, mammalian oocytes produce a large number of ribosomes along with other material needed to support their rapid growth. Once they reach their full size, oocytes can progress into meiosis. However, full sized oocytes display two distinct nuclear morphologies marked by a “surrounded nucleolus” (SN) or a “non-surrounded nucleolus” (NSN). Both types of oocytes can undergo meiosis and undergo fertilization, but NSN type oocytes are transcriptionally active whereas SN type oocytes are not. The nucleoli of SN oocytes undergo a distinct morphological change to form structures known as nucleolus-like bodies (NLBs). As oocytes undergo meiosis, the nuclear envelop breaks down resulting in the release of NLB components into the cytoplasm. More recent work has shown that NSN and SN oocytes can be easily distinguished from one another through use of a Fibrillarin (FBL) GFP reporter (Wang and Na, 2021). In addition, NSN oocytes appear to experience more DNA damage compared to their SN counterparts based on γH2AX staining (Wang and Na, 2021). Further experiments will be needed to more fully characterize these differences. Strikingly, NSN oocyte derived embryos arrest at the two-cell stage of embryogenesis, whereas SN oocyte derived oocytes maintain a greater competence to complete embryogenesis. Together, these findings indicate that the regulation of the nucleolar morphology and activity are essential for the generation of competent oocytes.

### Ribosomal Protein Heterogeneity in Germ Cells

Accumulating evidence suggests that ribosomes within a given cell may be heterogeneous (Xue and Barna, 2012; Barna, 2015; Shi and Barna, 2015; Shi et al., 2017; Genuth and Barna, 2018; Leppek and Barna, 2019; Leppek et al., 2020). This heterogeneity can take on many forms including the differential post-translational modification of ribosome proteins and/or rRNA and differential ribosome protein composition. Differences in ribosomes have been hypothesized to promote distinct mRNA translation programs during development and in times of stress. However, the functional significance of ribosome heterogeneity within any one context should be carefully evaluated in light of findings that changes in overall ribosome levels can have differential effects on mRNAs that experience high or low rates in translation initiation (Mills and Green, 2017).

The *Drosophila* genome encodes several ribosomal protein paralogs (Marygold et al., 2007), which exhibit different expression patterns, providing a potential experimental platform for studying ribosome heterogeneity. Microarray analysis (Kai et al., 2005) and later RNA-seq experiments (Graveley et al., 2011), showed that several of ribosomal protein paralogs, including *RpS5b*, *RpS19b*, and *RpS10a*, display enriched expression in gonads. Most of the ubiquitously expressed RP paralogs are on the X chromosome, while the paralogous genes that exhibit tissue specific expression are on an autosome. These ribosomal protein paralogs may carry-out tissue specific functions. For example, mutations in *RpS5a* result in a minute phenotype and lethality, while deletion of *RpS5b* leads to female sterility (Kong et al., 2019; Jang et al., 2021). Transgenic rescue experiments suggest that *RpS5a* and *RpS5b* may serve partially redundant functions in the germline (Kong et al., 2019; Jang et al., 2021), but pulldown experiments suggest that RpS5b containing ribosomes may show a preference for engaging with mRNAs encoding factors in mitochondrial electron transport (Kong et al., 2019).

*RpL22-like* encodes alternative protein isoforms (L22-like and L22-like short), both of which are expressed in the gonad and incorporated into polysomes (Kearse et al., 2010). RpL22, but not RpL22-like, is SUMOylated, especially in testis and sumoylated RpL22 does not associate with ribosomes (Kearse et al., 2013). This suggests that RpL22 protein may function outside the context of translation, similar to how phosphorylation of RPL13a controls whether this protein associates with the ribosome or negatively regulates translation in an extra-ribosomal manner (Mazumder et al., 2003). The functional role of SUMOylated RpL22 biological function should be investigated more thoroughly during germ cell development. Like RpS5a and RpS5b, RpL22 and RpL22-like appear functionally redundant within germ cells. Interestingly, expression of RpL22 results in decreased levels of RpL22-like, and vice versa, suggesting both proteins regulate the expression of one another to achieve a specific level of RpL22/RpL22-like within germ cells (Mageeney et al., 2018).

In contrast to *Drosophila* and several other model systems, few ribosome protein paralogs exist in mammals. However, some of these may carryout germline specific functions. For example, RpS4 paralogs have been shown to be differentially expressed in male and females. *RpS4X* and *RpS4Y* genes are located on X and Y chromosomes, respectively, and their dysfunction is linked with Turner syndrome (Fisher et al., 1990). RpS4X and RpS4Y differ by 19 amino acids, and both proteins appear interchangeable based on the complementation of temperature sensitive *RpS4X* mutant cells (Watanabe et al., 1993). Furthermore, proteomic analysis of cells from the liver, mammary gland, and testis revealed that paralogs of RpL10 and RpL39, referred to as RpL10-like and RpL39-like, exhibit specific expression in the testis (Sugihara et al., 2010). Future work will be needed to assess the extent to which these gonad specific ribosome protein paralogs function in a tissue specific manner.

### Germ Cell Specific Translation Initiation and Elongation Factors

Germ cells also express specific paralogs of broadly used translation factors. For example, the *Drosophila* genome contains eight eIF4E paralogs (Hernández et al., 2005), some of which exhibit specific enriched expression within gonads. The expansion of the number of eIF4 complex members and the germline-specific expression of individual paralogs may provide a sophisticated network of interactions for controlling mRNA translation in space and time within developing germ cells. Along these lines, the *Drosophila* testis expresses relatively high levels of eIF4E-3 and eIF-4 gamma and low eIF5B (Graveley et al., 2011). eIF4E-3 and eIF4G2 are both essential in male fertility and eIF4G2 is needed to drive the germline expression of CycB and Cdc25, both of which are important in meiosis (Hernández et al., 2005; Baker et al., 2015; Ghosh and Lasko, 2015). In addition, a mutation in eIF4G3 also results in male infertility in mice, marked by a failure of spermatocytes to exit meiotic prophase (Sun et al., 2010). Loss of the CDC2A kinase chaperone HSPA2 leads to strikingly similar phenotypes. Further experiments showed the eIF4G3 mutants failed to express HSPA2 protein despite the presence of *Hspa2* mRNA within these cells. These observations indicate that eIF4G3 mediates the translation of specific messages needed for meiotic exit. Interestingly, a subsequent study showed that eIF4G3 and several other components of the translation machinery localize to the XY body, a chromatin domain formed by transcriptionally inactive sex chromosomes (Hu et al., 2018). These observations suggest that spermatocytes may employ different germ cell-specific mechanisms for regulating the availability of translation factors needed for progression through meiosis.

The *C. elegans* genome also encodes at least five eIF4E-like genes, the function of which have recently been reviewed by Huggins and Keiper (2020). A number of these eIF4E isoforms play important roles in germline maintenance and development (Huggins et al., 2020). For example, IFE-1 exhibits enriched expression in germ cells and the protein associates with P granules. Mutations in IFE-1 result in fertility problems, including both reduced translation of specific maternally deposited mRNAs and defects in sperm development. Mutations in another eIF4E gene, IFE-3, result in defects in growth and germline sex determination. More specifically, the transition from spermatogenesis to oogenesis appears disrupted in IFE-3 mutant hermaphrodites. This defect can be suppressed by disrupting a key masculinizing gene, *fem-3*. IFE-3, along with its binding partner IFET-1, regulates the translation of several germline sex determination factors. By contrast, IFE-1 associates with PGL-1 and appears to regulate the expression of an independent set of mRNAs. The specificity of IFE-1 and IFE-3 function is mediated, at least in part, by association with their respective binding partners.

In addition to tissue specific initiation factors playing key roles in germ cell development and function, additional studies suggest germ cells in specific species may also employ specialized elongation machinery. *Xenopus* have three eEF1A genes. eEF-1S is expressed in the soma but not in germ cells, eEF-1O is expressed during oogenesis and in some adult tissues, and 42Sp50 is only expressed during oogenesis (Abdallah et al., 1991). Understanding the significance of this specialization amongst eEF1A paralogs and whether they drive different rates of elongation and/or influence overall translation fidelity will require further genetic interrogation. Interestingly, more recent work indicates that limiting eEF1A levels is likely an important control point in regulating the activity of germ cells. Work from the Wessel lab shows that protein synthesis rates within sea urchin PGCs is maintained at very low levels relative to neighboring somatic cells (Oulhen et al., 2017). This quiescent state in sea urchin PGCs is dependent on Nanos-2, which excludes eEF1A from PGCs (Oulhen et al., 2017). In addition, cytoplasmic pH has a marked effect on translation rates within PGCs. Similarly, *Drosophila* oocytes undergo extended periods of metabolic quiescence (Sieber et al., 2016). While much effort has gone into understanding the regulation of translation initiation in various contexts, germ cells may employ multiple modes of modulating mRNA translation, including cell-specific mechanisms for controlling elongation rates, to achieve a quiescent state. Further work will be required to determine whether the dynamic regulation of translation elongation represents a commonly used mechanism to control germ cell activity and quiescence.

### Communication Between Somatic Cells and Germ Cells Influences mRNA Translation

The maintenance and development of germ cells depends on local communication with their somatic cell neighbors. Long-range and systemic signals also influence germ cell activity. Work in *Drosophila* has illustrated how signals from fat tissue can modulate mTOR signaling within germline stem cells (Armstrong et al., 2014). Subsequent studies from the Drummond-Barbosa lab have continued to characterize how interorgan communication influences germ cell development in flies (Matsuoka et al., 2017; Armstrong and Drummond-Barbosa, 2018; Weaver and Drummond-Barbosa, 2018, 2019, 2020, 2021). Work from the Conti and Eppig labs, among others, shows that bidirectional communication between developing oocytes and their somatic cell neighbors also plays an important role in mammalian germ cell development (Chen et al., 2013; Wigglesworth et al., 2013). This signaling often converges on mRNA translation and the translation of maternal messages is enhanced in the presence of specific somatic cells (Chen et al., 2013). Additional studies show that FSH regulates mRNA translation in mouse oocytes, through indirect mechanisms involving EGF signaling within follicular cells (Franciosi et al., 2016; Tetkova et al., 2019). Signaling through the mTOR pathway acts as a key regulator of mouse germ cell development. For example, the survival of cumulus-oocyte complexes (COCs) depends on mTOR activation (Guo et al., 2016). Paracrine signaling from the oocyte suppresses a negative regulator of mTOR activity within the cumulus cells. In turn, mTOR activation within these cells controls the survival and differentiation of COCs. Conditional loss of *mTOR* in primordial or growing oocytes also causes infertility (Guo et al., 2018), marked by reduced translation of various mRNAs including *protein regulator of cytokinesis 1* and disruption of the first meiotic division. Interestingly, a population of transcripts, many of which play roles in meiotic progression, remain stored within the nuclei of oocytes during their early development (Susor et al., 2015). Upon nuclear envelope breakdown during the first meiotic division, these transcripts, which remain closely associated with chromatin, are translated in an mTOR and eIF4F dependent manner. mTOR activation leads to the phosphorylation and inactivation of 4E-BP1, a well-characterized inhibitor of cap-dependent translation. Work from other groups show that the temporally regulated translation of cell cycle genes, including *Cyclin B2*, helps to drive the progression of meiosis in mouse oocytes (Daldello et al., 2019). Experiments designed to further characterize how intercellular signaling influences mTOR activity, and global and transcript-specific translation represent important work in the coming years. Further insights into this regulation will likely yield improved methods for promoting germ cell differentiation and extended oocyte culture *in vitro*.

### Ribosome Accumulation and Storage in Oocytes

Studies dating back to the 1960’s observed that protein synthesis in mammalian embryos starts before zygotic transcription is initiated, indicating that maternally loaded and stored ribosomes are essential for early embryonic development. Indeed, work using *C. elegans* shows that maternally loaded ribosomes can support embryonic development from fertilization until the first larval stage, a time encompassing many cell divisions and tissue diversification (Cenik et al., 2019). The ability of oocytes to store vast quantities of active ribosomes may be a common feature across species. Previous electron microscopy studies revealed that mammalian oocytes store the majority of their ribosomes in cytoplasmic lattice-like (CPL) structures (Burkholder et al., 1971; Bachvarova et al., 1981). Whether similar structures exist in other species remains unexplored. More recent work indicates that the CPL also helps to coordinate organelle dynamics and the microtubule cytoskeleton within oocytes (Kan et al., 2011). Biochemical experiments suggest that the vast majority of ribosomes do not engage in translation during ovulation (Bachvarova and De Leon, 1977), further supporting the model that ribosome association with the CPL stores them in an inactive state. Genetic approaches are beginning to provide insights into the functional significance of CPLs. The ability of ribosomes to associate with the oocyte CPL is regulated by peptidylarginine deiminase 6 (PADI6) (Esposito et al., 2007; Yurttas et al., 2008). Loss of PADI6 results in infertility, marked by defects in protein synthesis and defective embryonic gene activation at the two-cell stage. The CPL cannot be visualized in PADI6 mutants. These results suggest that ribosome association with the CPL is critical for normal mRNA translation during early embryogenesis. Further biochemical studies identified components of the subcortical maternal complex (SCMC) including FLOPED, MATER, FILIA, and TLE6. FLOPED, MATER, and TLE6 are maternally deposited and interact with one another, while FILIA only interacts with MATER (Li et al., 2008). MATER co-localizes with PADI6 within the CPL of mouse oocytes, and loss of MATER results in infertility, marked by developmental arrest in two-cell embryos, similar in many ways to the phenotype caused by loss of maternal PADI6. Loss of MATER also disrupts the distribution of the endoplasmic reticulum and Ca^2+^ homeostasis (Kim et al., 2014), indicating that the protein has functions beyond ribosome storage. The similarity in the developmental arrest phenotypes of NSN oocyte derived embryos with those derived from PADI6 and MATER mutant oocytes is striking. While most antral oocytes from wild-type controls exhibit an SN morphology, 84% of oocytes from a MATER homozygous hypomorphic mutant display a NSN phenotype, suggesting a close connection between oocyte nucleoli, CPLs, and ribosome activity with developmental competence (Monti et al., 2013). However, more recent work suggests that cytoplasmic lattices are not linked with the developmental arrest of two-cell embryos (Longo et al., 2018).

### Breakdown of mRNA Translation in Reproductive Aging

Female mammals are born with a finite complement of oocytes. Thus, the female reproductive system begins to age before most other organs. In humans, female reproductive aging is marked by a decline in egg quality, starting late in the third decade of life, and progresses to complete loss of fertility by the time of menopause (Broekmans et al., 2009). Advanced reproductive age is marked by an increase of miscarriages and birth defects (Jones and Lane, 2013). These problems are most readily attributable to gametes: the majority of maternal age effects normally observed in older females are negated when eggs from young healthy donors are used in IVF procedures (Check et al., 2011).

The quality of eggs depends on maternally produced components including mRNAs, proteins, and organelles needed for the completion of early embryogenesis. Human oocytes can remain quiescent for over 40 years, and emerging evidence indicates that older eggs experience a decline in their ability to carry out mRNA translation (Duncan et al., 2017; Duncan and Gerton, 2018). Similar observations have been made in a broad range of species, including mice and *Drosophila* (Duncan et al., 2017; Greenblatt and Spradling, 2018; Greenblatt et al., 2019). Despite these observations across the animal kingdom, we still do not understand the basis for this decline in mRNA translation. Potential causes include, but are not limited to, reduced levels of ribosomes, reduced levels of tRNAs, reduced levels of initiation factors, and/or reduced levels of elongation factors. In addition, several recent papers using worms and flies have shown protein aggregation negatively impacts gamete production (Burn et al., 2015; Bohnert and Kenyon, 2017).

*Drosophila* has emerged as a useful model for studying the changes in mRNA translation that occur with age. Recent results have shown that the quality of *Drosophila* eggs declines the longer they remain stored and unfertilized within females (Greenblatt and Spradling, 2018; Greenblatt et al., 2019), mimicking what happens in other species such as mammals. Using Ribo-seq, Greenblatt and Spradling (2018) found that stored *Drosophila* eggs experience a decrease in mRNA translation. This decrease is accompanied by a loss of meiotic spindle components and a failure to support viable embryos, again consistent with what has been described in mammals (Duncan et al., 2017; Duncan and Gerton, 2018).

The underlying basis of the decline in mRNA translation in stored eggs across species remains poorly characterized. One possibility is that ribosome levels and function decline with age. The half-life of a typical ribosome within somatic cells is on the order of days. By contrast, eggs, which are stored in a transcriptionally quiescent state and therefore do not produce new rRNA, must maintain the same pool of ribosomes over the course of weeks, months, years, or even decades, depending on the species. Perhaps the ability of “old” ribosomes, which have potentially participated in multiple rounds of translation, to efficiently translate mRNAs of genes involved with regulating the meiotic spindle declines with age. It will also be interesting to evaluate whether the CPL breaks down over long periods of storage in mammalian oocytes.

### Common Themes and Unanswered Questions

Germ cells across species rely on a complex network of mRNA binding proteins to regulate translation in space and time. These networks extend beyond Vasa, Nanos, and Pumilio, and our knowledge regarding how RNA binding proteins regulate germ cell formation and function remains far from complete. The comprehensive characterization of these networks and understanding how they interact with each other and with germ cell specific translation machinery, at a systems level, remains critical work for the future. Perhaps more significantly, we are just beginning to understand how RNA binding proteins that carry intrinsically disordered regions govern germ granule formation. Moreover, recent work has revealed spatial organization within germ granules, adding to the complexity of the system (Trcek et al., 2015, 2020). Understanding the biophysical properties of these condensates, what governs their formation and dissolution, how the movement of different RNAs and proteins in and out of these structures is controlled, and how these granules contribute to and depend on both *cis*- and *trans* regulation of mRNA translation all represent important goals for the field in the coming years. This work will have a broad impact across multiple fields.

In addition, observations made across multiple species indicate that germ cells regulate ribosome biogenesis in a stage specific manner. Typically, robust positive correlations between ribosome levels and mRNA translation levels exist in somatic cells. However, this correlation does not always hold true in germ cells, whether in the context of early germ cell differentiation in *Drosophila* ovaries or quiescent vertebrate oocytes that store an enormous number of ribosomes. Germ cells across species often express germ cell specific paralogs of key translation factors, including ribosomal proteins. Whether these paralogs simply serve to increase overall levels of a general activity or carry out a highly specialized function largely remains an open question. For example, do ribosomes that carry germ cell specific ribosome protein paralogs target specific messages for translation or exhibit different behaviors such as different rates of translation elongation or fidelity? Recent work in *Drosophila* has failed to detect clear functional differences between RpS5A and RpS5B paralogs. However, these experiments were carried out in a lab setting and not out in the wild. Perhaps, functional differences between general and germ cell enriched translation factors will only become apparent under the appropriate environmental conditions. Lastly, the mechanisms that control ribosome activity and storage within the germline are also just coming into focus. How are ribosomes stored for long periods of time? Are there functional differences between maternal and zygotically produced ribosomes? Can manipulating ribosome levels or activity prolong reproductive aging? Further insights into these areas will enhance our understanding of reproductive biology. Thus, the study of mRNA translation within germ cells promises to remain an important area of study for the foreseeable future.

## Author Contributions

MB, MM, SJ, and CN contributed to the writing and editing of the review. All authors contributed to the article and approved the submitted version.

## Conflict of Interest

The authors declare that the research was conducted in the absence of any commercial or financial relationships that could be construed as a potential conflict of interest. The reviewer PR declared a past co-authorship with the author MB to the handling editor.

## Publisher’s Note

All claims expressed in this article are solely those of the authors and do not necessarily represent those of their affiliated organizations, or those of the publisher, the editors and the reviewers. Any product that may be evaluated in this article, or claim that may be made by its manufacturer, is not guaranteed or endorsed by the publisher.
